# Effect of Culturally Adapted Dental Visual Aids on Anxiety Levels in Children with Autism Spectrum Disorder: A Randomized Clinical Trial †

**DOI:** 10.3390/children10061040

**Published:** 2023-06-09

**Authors:** Ala Aljubour, Medhat AbdElBaki, Omar El Meligy, Basma Al-Jabri, Heba Sabbagh

**Affiliations:** 1 Pediatric Dentistry Department, Faculty of Dentistry, King Abdulaziz University, Jeddah 21589, Saudi Arabia; eabdulbaqi@kau.edu.sa (M.A.); omeligy@kau.edu.sa (O.E.M.); hsabbagh@kau.edu.sa (H.S.); 2 Pediatric Dentistry and Dental Public Health Department, Faculty of Dentistry, Alexandria University, Alexandria 21521, Egypt; 3 Pediatrics Department, Faculty of Medicine, King Abdulaziz University, Jeddah 21589, Saudi Arabia; baljabri@kau.edu.sa

**Keywords:** autism spectrum disorder, anxiety, child, visual aids, Saudi Arabia

## Abstract

Autism spectrum disorder (ASD) prevalence has risen dramatically in recent decades; it is now common for children with ASD to seek dental care. Because their distinct behavioral patterns prevent them from receiving dental care in a traditional dental clinic, structured dental visual aids are required to help prepare them for their dental appointment. This study aimed to test the effect of “culturally adapted dental visual aids” in decreasing anxiety levels during dental visits in children with autism spectrum disorder (ASD). A randomized, controlled, blinded clinical trial was performed. Sixty-four children with ASD ages 6–12 years were assigned randomly into test and control groups based on the type of dental visual aids they received. The test group received “culturally adapted dental visual aids” created especially for the study, and the control group received “regular dental visual aids”. Anxiety levels were assessed before and after using both dental visual aids. All data were processed using SPSS version 25.0. The test group had a significant decrease in anxiety levels compared to the control group (*p* < 0.001). The “culturally adapted dental visual aids” have effectively reduced anxiety levels in children with ASD during dental visits.

## 1. Introduction

Autism spectrum disorder (ASD) is a phrase that refers to a neurological and developmental disorder that has emerged to be a serious public health concern in several countries in recent years, usually commencing before age three [1]. The typical triad signs of ASD include severe deficiencies in social skills, linguistics, and strict repetitive activities [1]. When confronted with a new procedure, children with ASD experience increased levels of anxiety. It is of significant importance to provide them and their parents with preparatory material to assist them through the dental appointment, explaining each dental procedure step by step to reduce their anxiety levels.

Structured visual aids are an effective way of guiding children with ASD through their daily routine and teaching them new activities at home and school; they are made of a set of cards with drawings on them, where each one explains a daily routine or a dental visit step [2]. The main objective of dental visual aids is to prepare children with ASD for dental appointments, facilitate communication with the dental team, and reduce anxiety levels before the dental visit, thus promoting a positive attitude toward the dental appointment [3]. This approach allows patients with ASD to receive optimal dental treatment with the least anxiety and fear. In that way, psychological desensitization occurs, resulting in a significant reduction in anxiety levels and improved oral health [4,5].

Isong et al., in 2014, tested the impact of these aids on anxiety levels among children with ASD. Their findings suggested that some electronic screen media could be beneficial strategies for reducing anxiety levels during dental appointments in children with ASD [3]. Mah and Tsang, in 2016, investigated levels of anxiety among children with ASD by using an Electrodermal Activity device (EDA) inserted in a wristband on the patient’s right wrist for the duration of the dental session to detect physiological arousal [6]. They found that the group that received a picture version of dental visual aids had lower scores than the group that received Tell-Show-Do (TSD) only [6], which is a technique used frequently by pediatric dentists for behavior management during dental treatment that involves verbal explanations of the dental procedure in age-appropriate words (tell); demonstrations of the procedures (show); and then, without deviating from the explanation and demonstration, completion of the procedure (do) [7]. 

Eliminating anxiety in children with ASD during dental appointments is difficult, even when performing a simple dental procedure. Another method that has been proven effective in reducing the anxiety of children with ASD is the use of deep pressure, as described by Afifi et al. in 2022 [8,9].

Dental visual aids are commonly used with such children to help reduce their anxiety levels. Providing customized dental visual aids in the child’s local language has proven its efficacy in decreasing the child’s anxiety and improving compliance [10]. However, the available versions are not illustrated in our local Arabic language and do not reflect our society. There is a need to find an approach that represents our society, to reduce levels of anxiety among children with ASD. Not many studies have been performed in the literature to address such an important matter that affects the quality of dental treatment provided to children with ASD. Therefore, our research aimed to test the effect of “culturally adapted dental visual aids” in decreasing levels among children with ASD during dental visits.

## 2. Materials and Methods

### 2.1. Ethical Approval

Ethical approval was obtained before conducting the research from the Research Ethics Committee at the Faculty of Dentistry in King Abdulaziz University (KAU), Jeddah, Saudi Arabia, under approval number 057-02-19. Consent forms were signed by the parents for the participation of their children in the study (Appendix A). 

### 2.2. Sample Size

By using the OpenEpi program, version 3, a sample size of 64 children with ASD divided into two groups, with 32 children in each group, was calculated to have a confidence interval of 95 percent and a power sample of 80 percent based on prior research in the literature [11].

### 2.3. Participants’ Eligibility Criteria 

All children attending King Abdulaziz University Hospital’s (KAUH) ASD diagnosis clinic from January 2019 to January 2021 were encouraged to participate in the research. Diagnosis of ASD was confirmed in the files of children who met the inclusion criteria [1]. Interviews with the parents and the children and hospital records were used to supplement the data.

### 2.4. Inclusion Criteria

The inclusion criteria were as follows: (1) Saudi Arabian patients; (2) 6–12 years old patients; (3) a confirmed ASD diagnosis by a specialized professional in the field; (4) patients with no dental experience, (5) patients who, based on the “Childhood Autism Rating Scale” (CARS), have mild to moderate ASD [12].

### 2.5. Exclusion Criteria

Patients with congenital abnormalities, such as Down syndrome and cerebral palsy.

### 2.6. Study Design

A double-blinded, randomized, controlled clinical trial.

### 2.7. Blinding and Randomization

At the beginning of the study, a total of 64 children with ASD (43 males and 21 females) were randomly divided into two groups depending on the type of dental visual aids they received using the simple randomization technique of tossing a coin by the observer, with heads indicating group I (test group) and tails indicating group II (control group). On an Excel file generated for blindness, each participant received a different serial number that corresponded to the group to which they were assigned. The principal investigator and an observer conducted the study, with the principal investigator performing all the dental procedures. After the first dental assessment, the principal investigator and the observer were blinded to the patient’s allocation group. In the second dental assessment visit, the observer made a new set of documents utilized for each patient with an assigned serial number but no mention of the type of dental visual aids used. In addition, the statistician was blinded to the data.

### 2.8. Interventions (Dental Visual Aids)

**Figure 1:**
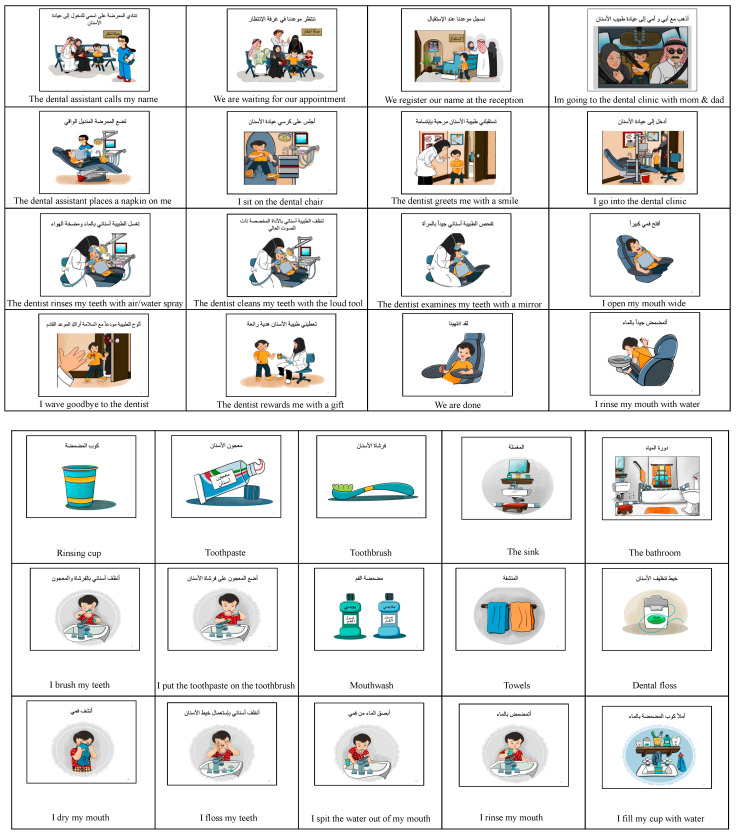
The culturally adapted dental visual aids: dental clinic and oral hygiene instructions.

Group I received the “culturally adapted dental visual aids” explicitly made for this study to fit the culture of Saudi society. They were created by recruiting a specific artist that designed and drew colored graphics based on pictures taken of the pediatric dentistry clinics at King Abdulaziz University Dental Hospital (KAUDH). Three ASD specialists, including an ASD-specialized physician, a child psychologist, and a behavioral psychologist, verified the “culturally adapted dental visual aids”. Moreover, before starting the study, an Arabic language expert checked the Arabic language, as illustrated in Figure 1.

**Figure 2:**
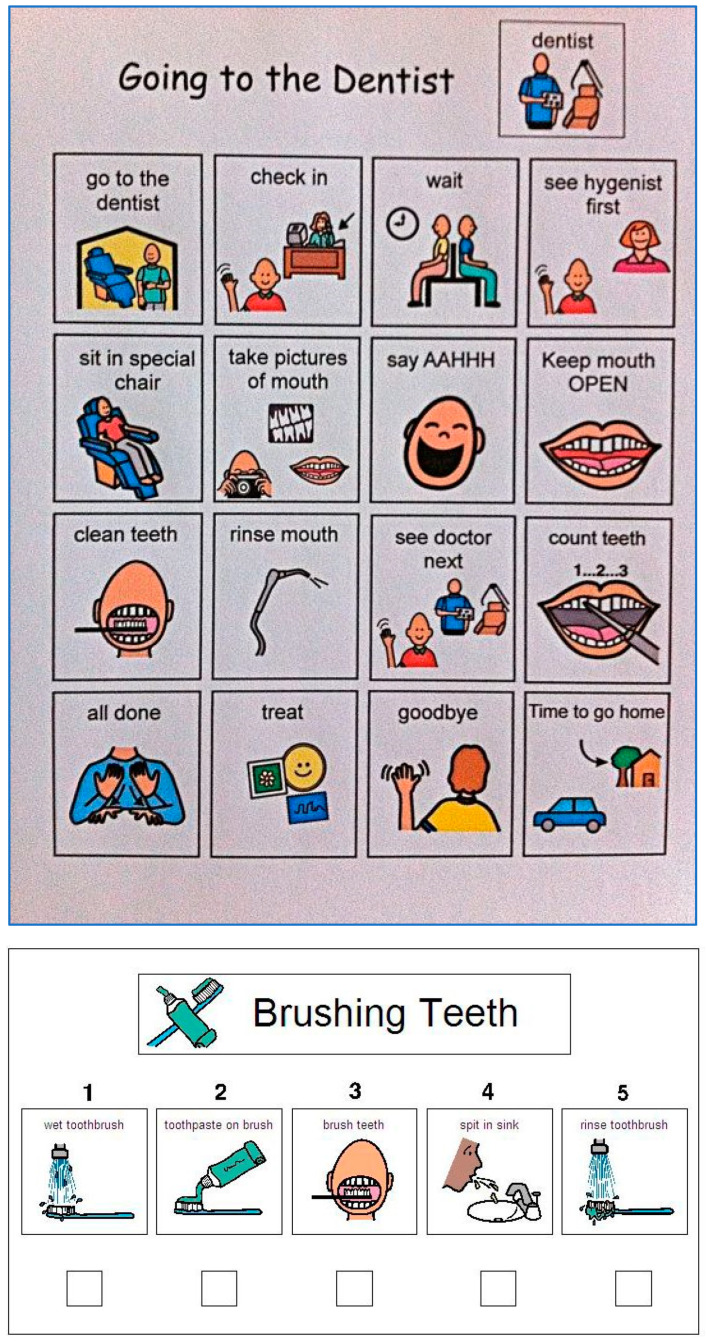
The regular dental visual aids: dental clinic and oral hygiene instruction.

Figure 2 demonstrates the “regular dental visual aids” given to group II, obtained from www.ageofautism.com (accessed on 4 March 2019). They were retrieved in their original English language and described verbally in the Arabic language for the parents and the children. 

The principal investigator and the parents in both groups agreed on a selection of phrases to be explained to the children at home in Arabic daily for four weeks.

### 2.9. Preparatory Visit 

The first visit took place at the ASD diagnosis clinic in KAUH, which was a preparatory visit for the parents, where the principal investigator explained the nature of the study to them. Afterward, the principal investigator interviewed each guardian to answer a questionnaire that had two parts; the first part inquired about the child’s age, gender, and the parent’s socioeconomic status derived from the “Central Department of Statistics and Information in the Kingdom of Saudi Arabia” (CDSIKSA) [13], and the second part inquired about each child’s medical and dental history. One week later, children and their parents were invited to start the dental assessment visits at the pediatric dentistry clinics in KAUDH.

### 2.10. Pre-Test Questionnaire

Ten random parents were selected to pre-test the demographic questionnaire to ensure it was being clearly understood and interpreted. Adjustment and rephrasing of the questions were later performed to make them easily understood. 

### 2.11. First Dental Assessment Visit

The parents in both groups were given the “Anxiety Scale for Children-Autism Spectrum Disorder” (ASC-ASD) [14] to fill out, which is a self-administered questionnaire directed to the parents inquiring about their child’s anxiety levels (Appendix B). This questionnaire is a validated questionnaire used to assess anxiety in children with ASD, which was translated and validated in both English and Arabic languages [14]. Upon answering the questions on the anxiety scale, the child began the first dental assessment visit; the only behavior management technique used with the children during this visit was TSD. A single calibrated principal investigator performed all dental procedures. 

The first dental assessment visit included the following: (1) an oral exam, (2) plaque index scores measurements [15], (3) professional prophylaxis using a small rotary cup with non-fluoridated paste on a low-speed handpiece, and (4) oral hygiene instructions given to the parents and children explained on a plastic dental cast. Each child received a hard copy of the dental visual aids at the end of the visit, according to their allocation group, which were demonstrated to them in Arabic. 

Parents were instructed to read the dental visual aids daily to their children for at least fifteen minutes at the same time every day [16] for a minimum duration of four weeks. The observer called the parents of both groups two weeks later to ensure that parents adhered to the instructions and that the dental visual aids were being read daily to the children.

### 2.12. Second Dental Assessment Visit

Participants were called four weeks later for the second dental assessment visit. The TSD technique was the only behavior management approach used with the children during this visit. All dental procedures were performed by the same calibrated principal investigator, and the same observer recorded the plaque index scores implemented by the principal investigator.

In the second dental assessment visit, the dental procedures were as follows: (1) an oral exam, (2) plaque index score measurements [15], (3) professional prophylaxis using a small rotary cup with non-fluoridated paste on a low-speed handpiece. The parents were asked to fill out the anxiety scale; ASC-ASD [14]. The answers from both visits were later compared to determine how effective the dental visual aids are in reducing anxiety levels in children with ASD. Children who needed additional treatment were referred to the postgraduate department for comprehensive dental treatment at KAUDH’s pediatric dentistry clinics.

### 2.13. Anxiety Level Scale

This scale contained 24 items inquiring about anxiety; each question was scored using a four-point Likert scale ranging from zero to three. The results were calculated by adding the numerical values from the answers, where 0 = Never, 1 = Sometimes, 2 = Often, and 3 = Always. The lowest score possible was 0, meaning the child had no anxiety at all, and the highest score possible was 72. An anxiety total from 1 to 19 indicated that the child had anxiety, and a score of 20 or more indicated that the child had high levels of anxiety. In the case of any missing answers, the numerical value of that question was prorated within the remaining questions. 

The scale contained four anxiety subscales, including (1) “Separation Anxiety”, (2) “Uncertainty”, (3) “Performance Anxiety”, and (4) “Anxious Arousal”. The existence of that specific anxiety subcategory was determined by adding the numerical values of specific questions from the anxiety scale, with the total sum of these questions indicating the presence of that specific anxiety subcategory. There are no cutoffs while calculating the subscales; either the child had or did not have the subscale anxiety. 

The numerical values of five items, including the following questions: 2, 4, 7, 15, and 17, were added to determine whether the child had any performance anxiety. The minimum value was 0, indicating that the child had no performance anxiety at all, and the maximum value was 15, indicating that the child had performance anxiety.

The numerical values of six items, including the following questions: 1, 3, 8, 12, 13, and 22, were added to determine whether the child had any anxious arousal. The minimum value was 0, indicating that the child had no anxious arousal, and the maximum value was 18, indicating that the child had anxious arousal.

The numerical values of five items, including the following questions: 11, 18, 19, 20, and 24, were added to determine whether the child had any separation anxiety. The minimum value was 0, indicating that the child had no separation anxiety at all, and the maximum value was 15, indicating that the child had separation anxiety.

The numerical values of eight items, including the following questions: 5, 6, 9, 10, 14, 16, 21, and 23, were added to determine whether the child had any uncertainty. The minimum value was 0, indicating that the child had no uncertainty, and the maximum value was 24, indicating that the child had uncertainty.

### 2.14. Calibration and Reliability

By selecting 10 charts, the completeness of the information in the assessment charts was evaluated at random by a third clinician with competence in ASD research. There was an inter-examiner agreement of 100%.

### 2.15. Statistical Analysis

The Statistical Package for the Social Sciences (SPSS) version 25.0 (IBM Inc., Chicago, IL, USA) was used to analyze the data. The descriptive statistics, frequency, percentage, and chi-square test were used to examine the demographic and clinical characteristics of the study participants. The confidence level was 95%, while the significance level was 0.05. The mean rankings within each group were compared using the Wilcoxon signed-rank test before and after using the dental visual aids, and the mean ranks between the two groups were compared using the Mann-Whitney test.

## 3. Results

**Figure 3:**
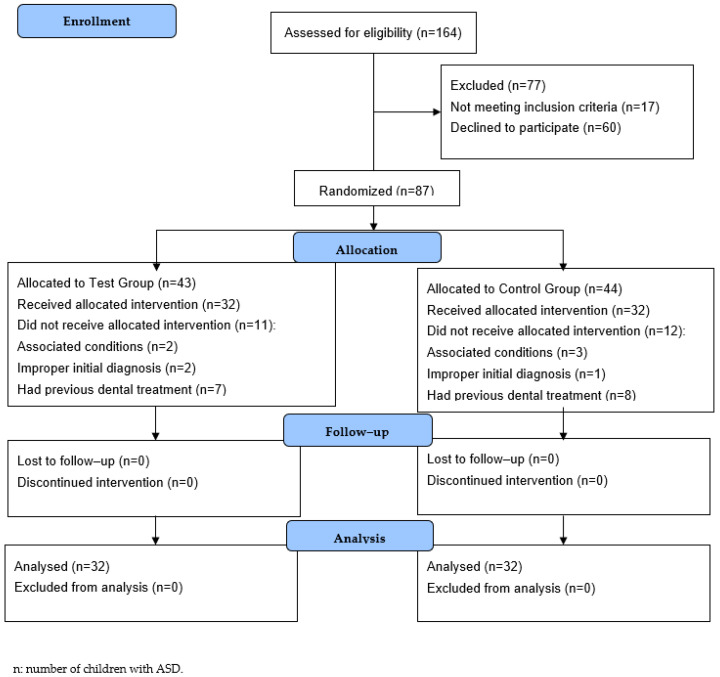
The CONSORT transparent reporting of trials.

A CONSORT diagram showing the study protocol is presented in Figure 3.

### 3.1. Characteristics of the Participants

The participants were Saudi children with ASD ages 6–12, with a mean age of eight years and two months. The distribution of gender among the groups was surveyed, and it was not statistically significant (*p* = 0.790).

The mean age of ASD diagnosis was 5.5 years. The severity of ASD in children was investigated, and it was not statistically significant (*p* = 0.209).

### 3.2. Anxiety Scale for Children with ASD

#### 3.2.1. Intragroup Comparison 

Using the ASC-ASD, anxiety scores in children with ASD, as reported by their parents, were measured before and after using the “culturally adapted dental visual aids” in the test group. The total anxiety mean was lower after using the “culturally adapted dental visual aids”, and it was statistically significant (*p* < 0.001); however, there was no statistically significant difference in the total anxiety mean in the control group after using the “regular dental visual aids” (*p* = 0.532). 

When comparing the anxiety subscales, we found that the mean in the test group was lower after using the “culturally adapted dental visual aids”, and it was statistically significant, whereas there was no statistically significant difference in the control group after using the “regular dental visual aids” in all the anxiety subscales. The total anxiety score was higher in the test group before the intervention; however, it was only higher in two subdivisions, as shown in Table 1.

#### 3.2.2. Comparison between the Groups 

To measure the effectiveness of the “culturally adapted dental visual aids” given to the test group in reducing anxiety levels in children with ASD, we compared differences in anxiety levels as reported by the parents before and after the use of dental visual aids between both groups. 

The test group had a lower mean value of total anxiety score than the control group (−8.25, 0.00), respectively, and it was statistically significant (*p* < 0.001). These findings were observed in all four subscales of anxiety levels, as seen in Table 2.

## 4. Discussion

We compared the effectiveness of the “culturally adapted dental visual aids” specially designed for the research in reducing levels of anxiety among children with ASD during dental visits. We observed that anxiety levels decreased significantly after using the “culturally adapted dental visual aids” in the test group compared to the control group. 

It has been reported that oral health care in children with ASD is often neglected due to their complex behavior patterns [17] and their increased anxiety levels, especially when faced with a new situation, such as visiting the dental clinic. Delivering optimal dental care for children with ASD requires considerable training and preparation. Therefore, dental visual aids are essential to lower their anxiety levels and have a positive attitude toward dental appointments [2].

Anxiety level measurement in children can be a complex process that depends on various factors. There are several ways to measure anxiety levels in children, including self-report questionnaires, behavioral observations, and physiological measures such as heart rate and cortisol levels. Self-report questionnaires are commonly used to assess anxiety levels in children. These questionnaires usually ask children to rate how anxious they feel in different situations or to answer questions about their thoughts and feelings related to anxiety. The responses are then scored and analyzed to determine the child’s anxiety level. It is important to note that the measurement of anxiety levels in children is not always straightforward and can be influenced by various factors, including the child’s age, developmental level, and cultural background. Additionally, anxiety is a complex emotion that can manifest in different ways, so it is important to use multiple methods of assessment to obtain a complete picture of a child’s anxiety level [18].

The present study developed “culturally adapted dental visual aids” that represented our culture and illustrated our actual dental clinic, dental team, and dental appointment steps, in addition to illustrating the oral hygiene instructions at home. We aimed to create a pictorial social story familiar to children with ASD that would reflect their society, culture, and language. Nilchian et al., in 2017, validated this theory by simulating the dental clinic and the actual dental team that a child with ASD would encounter, allowing them to become accustomed to the environment they will experience [10]. 

The children with ASD in the control group, on the other hand, were given dental visual aids obtained from www.ageofautism.com (accessed on 4 March 2019). They contained a series of pictures illustrating the dental appointment steps and the oral hygiene instructions at home in simple English words that are being used by children with ASD all over the world [6]. However, they are not commonly used in Saudi Arabia because of the language barrier. The “regular dental visual aids” were explained in Arabic to both the parents and the children with ASD to avoid any bias that could result from comparing two dental visual aids in different languages.

In the present study, 32.8% of the participants were girls, 67.2% were boys, and the male-to-female ratio was 2:1. These numbers are explained by the higher prevalence of ASD among males than females, which came in accordance with the numbers mentioned in the literature [19].

Our research focused on a sample of Saudi children aged 6–12 years who had ASD. This age group was chosen to start at six years old, as the literature has shown that children with ASD can have a good grip on a toothbrush for teeth brushing at that age [20], and end at 12 years old to eliminate the possibility of puberty hormones interfering negatively with their unique behavioral patterns [21].

Moreover, we selected children that had no previous dental experience, as having a previous traumatic event could negatively impact the behavior of the child as well as influence the ability to acquire a new skill proposed in the dental visual aids; this selection was made following a previous study [22].

Participants in this study were selected with mild to moderate ASD according to CARS [12]; this inclusion was based on the child’s ability to speak and engage in a straightforward conversation. In contrast, a child with moderate ASD can speak a few words but is unable to carry on a conversation, but they can comply with commands given to them during a dental appointment. Previous studies in the literature helped to justify the choice of these criteria [10].

Due to the inclusion of children with mild to moderate ASD, we used the TSD, which is a basic behavior management technique that was understandable for their cognitive level; the use of this approach agrees with Orellana et al. [23], who used the same approach in their research with children with ASD.

In order to effectively change any behavior in children with ASD and prepare them to learn a new skill, all parents in both groups were instructed to spend at least 15 min a day explaining the dental visual aids to the children for at least four consecutive weeks [16].

In the current study, levels of anxiety have reduced significantly among children with ASD after implementing the “culturally adapted dental visual aids”, compared to the control group. This decrease is in line with the findings of Mah and Tsang in 2016, who tested the effectiveness of dental visual aids on anxiety levels of children with ASD by measuring their physiological arousal during the dental visit using an Electrodermal Activity device (EDA) and found that EDA readings had a significant reduction in the picture dental visual aids group versus the group that had TSD only [6]. This agreement is explained by the similarity of the dental visual aids used in their research and ours (picture dental visual aids) and the preference for visual communication in children with ASD rather than verbal.

Moreover, Isong et al., in 2014, did not agree with our results, where they recorded anxiety levels using Venham Anxiety and Behavioral Scales in children with ASD during the dental appointment [3]. They measured their scores over two dental appointments 4–6 months apart. They observed a noticeable reduction in anxiety levels in two groups that had dental visual aids during the second dental appointment. On the other hand, there was no significant difference in anxiety scores in the other two groups that had video peer modeling only. This disagreement might be explained by the difference in the anxiety measuring scales between our study and theirs. Moreover, having a probe on the child’s finger throughout the dental appointment might have exacerbated the sensation sensitivity leading to a high anxiety score.

Aljubour et al., in 2021, published a systematic review that included all studies that tested the effectiveness of various dental visual aid types on children with ASD during dental appointments. They found that almost all studies in the review had shown successful results in improving the dental experience for children with ASD [24].

There are a few limitations to consider in this study. Children with severe ASD were not included in the study. The study was restricted to only Saudi children attending the KAUH ASD diagnosis clinic. Part of the study protocol was reliant on the parents’ adherence to reading the dental visual aids daily for four weeks to their children. Moreover, the use of picture version dental visual aids was not electronic.

## 5. Conclusions

Based on this study’s results, the following conclusion can be made: The culturally adapted dental visual aids have proven their efficacy in reducing anxiety levels in children with ASD during dental visits. Due to its effectiveness, this protocol could be used as future guidelines for decreasing anxiety levels in children with ASD before dental treatment. Future research on the effectiveness of electronic versions of the “culturally adapted dental visual aids” is suggested. 

## Figures and Tables

**Table 1:** Comparison between anxiety levels before and after using the dental visual aids within each group.

	Group		Mean	SD	MED	*p*-Value
Total Anxiety Score	Test	Before	22.090	11.650	19.500	<0.001 *
		After	13.840	8.600	13.500	
	Control	Before	18.060	10.540	17.500	0.532
		After	18.060	12.160	14.00	
Performance Anxiety	Test	Before	3.440	2.970	3.00	0.001 *
		After	1.940	2.200	1.00	
	Control	Before	3.000	3.210	2.500	0.259
		After	2.625	3.170	1.500	
Anxious Arousal	Test	Before	3.690	2.290	3.00	<0.001 *
		After	2.125	1.720	2.00	
	Control	Before	3.310	2.670	2.00	0.583
		After	3.500	2.910	2.500	
Separation Anxiety	Test	Before	6.720	4.200	7.00	0.002 *
		After	4.375	2.485	4.00	
	Control	Before	5.090	2.720	5.00	0.692
		After	5.030	3.050	5.00	
Uncertainty	Test	Before	8.250	5.435	8.500	<0.001 *
		After	5.440	4.370	4.00	
	Control	Before	6.660	4.190	6.00	0.522
		After	7.190	4.770	8.00	

SD: Standard deviation. MED: Median. *: Statistically significant *p <* 0.05. Wilcoxon signed-rank test.

**Table 2:** Comparison of anxiety levels between test and control groups for the value between before and after using the dental visual aids.

	Test Group	Control Group	*p*-Value
Anxiety Level	Mean	Mean	
Total Anxiety Score	−8.25	0.00	<0.001 *
Performance Anxiety	−0.15	−0.38	0.026 *
Anxious Arousal	−1.56	1.88	<0.001 *
Separation Anxiety	−2.34	−0.063	0.003 *
Uncertainty	−2.813	0.53	<0.001 *

*: Statistically significant *p <* 0.05. Mann-Whitney test.

## Data Availability

Data created or analyzed during this study is available upon request from the corresponding author.
